# Response to Comment on: Patient out-of-pocket costs for guideline-recommended treatments for erectile dysfunction: a medicare cost modeling analysis

**DOI:** 10.1038/s41443-024-00973-9

**Published:** 2024-09-23

**Authors:** Vi Nguyen, Darshan P. Patel, Tung-Chin Hsieh

**Affiliations:** UC San Diego Health, San Diego, CA USA

**Keywords:** Sexual dysfunction, Surgery, Urinary tract

Erectile dysfunction (ED) is a common condition with increasing prevalence in the aging population. However, only 10% of men seek medical attention for sexual problems, and consequently, 70% of men with ED do not receive adequate treatment [1, 2]. Barriers to ED treatment are multifactorial and may include lack of consideration by clinicians, patient embarrassment, and financial burden [3]. Currently, the American Urological Association (AUA) 2018 Guidelines do not include discussion regarding cost of ED treatment options [4]. Thus, we devised a Medicare cost model to conservatively estimate patient out-of-pocket (OOP) costs for the six treatment options recommended by the AUA, which include oral phosphodiesterase type 5 inhibitors (PDE5i; strong recommendation), inflatable penile prosthesis (IPP; strong recommendation), vacuum erection device (VED; moderate recommendation), intracorporal injections (ICI; moderate recommendation), intraurethral alprostadil (IA; conditional recommendation), and low-intensity extracorporeal shock wave therapy (ESWT; conditional/investigational recommendation) [5]. An annual IA prescription had the highest OOP cost ($4,022), followed by annual ICI prescription ($3,947), one ESWT treatment course ($3,445), IPP as an outpatient procedure ($1,600), annual PDE5i prescription ($696), and one VED unit ($213) [5]. However, there were several limitations to our model, which include abstraction of data from several sources, the conservative assumption that each patient would only trial one treatment option annually, and lack of generalizability of our findings to patients not covered by Medicare. Furthermore, as mentioned by Iwuala et al., our data was primarily abstracted from GoodRx, which is not directly representative of patient costs after insurance is billed, highlighting the need for greater price transparency from pharmaceutical companies and insurance firms [6].

Furthermore, our model does not account for the recent emergence of online prescription platforms and direct-to-consumer (DTC) care [7]. Factors that likely contribute to the rising popularity of this care model include both time and geographic convenience. In addition, as mentioned above, patients who perceive their medical problems to be shameful, such as men with ED, may prefer this service as it bypasses in person interaction with a provider [8]. Questionnaire-based studies have demonstrated that reasons for seeking ED treatments through DTC platforms include convenience (48%), discretion (23%), and shame (13%) [8]. Common referral sources to the DTC platforms included television (40%), Google (29%), and online media (19%) [8]. Given the wide variety of these services, it remains unknown the range of costs of prescriptions obtained online.

Despite U.S. health care costs ranking among the highest in the world, many physicians have limited insight into the costs and insurance coverage of the treatment options that they recommend [9, 10]. Cortese et al. highlighted the potential cost savings to be garnered from urologic drug price stewardship [11]. The authors demonstrated a potential savings of approximately $1.29 billion in 2020 if Medicare prices for generic urologic drugs were as low as those offered by the Mark Cuban Cost Plus Drug Company, a public benefit corporation centered on manufacturing and distribution of lower priced generic drugs [11]. Thus, we hope that our findings will, in fact, change clinical management of ED by increasing awareness into the cost variation amongst the different guideline-recommended treatments. While it is true that the most cost-effective options (oral PDE5i and IPP) also were the most strongly recommended therapies, we hope that our model will encourage providers to not only consider individual patient financial burden but also cost effectiveness from a broader high value care lens to ultimately improve shared decision making with patients who suffer from ED.
